# S160. INTERACTIONS BETWEEN BOTTOM-UP AND TOP-DOWN ATTENTION DURING WORKING MEMORY ENCODING: EVALUATION OF AN FMRI PARADIGM FOR THE STUDY OF COGNITIVE DYSFUNCTION IN SCHIZOPHRENIA

**DOI:** 10.1093/schbul/sby018.947

**Published:** 2018-04-01

**Authors:** Mishal Qubad, Catherine Barnes, Lara Rösler, Michael Schaum, Benjamin Peters, Michael Wibral, Andreas Reif, Robert Bittner

**Affiliations:** 1 University Hospital Frankfurt, Goethe University; 2 Goethe University Hospital Frankfurt; 3 University of Würzburg Germany

## Abstract

**Background:**

Patients with schizophrenia suffer from profound impairments of working memory and selective attention. These cognitive domains show a considerable overlap on both the behavioral and neurophysiological level. Importantly, selective attention appears to be crucial for the selection of information to be encoded into working memory. A number of studies have demonstrated that the efficiency of this “gatekeeper” function influences working memory performance. Furthermore, behavioural evidence indicates, that patients with schizophrenia have a specific deficit when required to suppress irrelevant but highly salient visual information during working memory encoding. Therefore, elucidating the neurophysiological mechanisms underlying the “gatekeeper” function of selective attention for working memory is highly relevant for understanding this deficit in schizophrenia. The aim of the current study was to investigate the neurophysiological correlates of encoding either salient or non-salient information in the presence of distractors of opposite saliency using functional magnetic resonance imaging (fMRI). Furthermore, we wanted to study the impact of additional top-down information guiding the selection of task relevant information.

**Methods:**

35 healthy volunteers underwent fMRI in a 3 T Siemens Trio scanner. During a change detection task four Gabor patches (two flickering and two non-flickering) with varying orientations were shown and participants had to memorise the orientations of the Gabor patches. A colored fixation cross was displayed before the stimuli either cueing two (predictive cue) or four (non-predictive cue) Gabor patch locations resulting in a 2 x 2 design of four conditions with the factors salience (flickering vs. non-flickering) and cue (predictive cue vs. non-predictive cue). During retrieval a single Gabor patch was displayed, and participants reported if the orientation was the same or had changed in that location. At the beginning of each block participants were instructed to either encode the flickering or non-flickering patches (targets) whose location could either be cued or uncued. In 80 % of trials, a target was probed during retrieval. Data analysis in Brain Voyager included standard data preprocessing. Additionally, a multiscale curvature driven cortex based alignment procedure was used to minimise macro-anatomical variability between subjects. Subsequently, functional data were analysed using a random-effects multi-subject general linear model (p<0.05, FDR corrected). Functional connectivity analysis was performed using Granger Causality Mapping.

**Results:**

Participants were able to preferentially encode task-relevant information in all four conditions. During encoding, they showed activation in a distributed network of fronto-parietal and visual areas. For salient compared to non-salient distractors, we observed increased functional connectivity between attention-related areas and extrastriate visual cortex. This difference was more pronounced for trials with a predictive compared to non-predictive cue.

**Discussion:**

We were able to map the cerebral networks responsible for determining the contents of working memory. The observed patterns of connectivity indicate that core regions of the frontal-parietal network involved in both working memory and selective attention play a crucial role in the filtering of information by modulating the processing of information in visual areas. Our current findings provide the basis for studying the neurophysiological underpinnings of the interaction between impairments of working memory and selective attention in schizophrenia.

